# Do Red Seaweed Nanoparticles Enhance Bioremediation Capacity of Toxic Dyes from Aqueous Solution?

**DOI:** 10.3390/gels8050310

**Published:** 2022-05-17

**Authors:** Abdallah Tageldein Mansour, Ahmed E. Alprol, Mohamed Ashour, Khaled M. A. Ramadan, Adnan H. M. Alhajji, Khamael M. Abualnaja

**Affiliations:** 1 Animal and Fish Production Department, College of Agricultural and Food Sciences, King Faisal University, P.O. Box 420, Al-Ahsa 31982, Saudi Arabia; aalhajji@kfu.edu.sa; 2 Fish and Animal Production Department, Faculty of Agriculture (Saba Basha), Alexandria University, Alexandria 21531, Egypt; 3 National Institute of Oceanography and Fisheries (NIOF), Cairo 11516, Egypt; microalgae_egypt@yahoo.com; 4 Central Laboratories, Department of Chemistry, King Faisal University, P.O. Box 420, Al-Ahsa 31982, Saudi Arabia; kramadan@kfu.edu.sa; 5 Department of Biochemistry, Faculty of Agriculture, Ain Shams University, Cairo 11566, Egypt; 6 Department of Chemistry, College of Science, Taif University, P.O. Box 11099, Taif 21944, Saudi Arabia; k.ala@tu.edu.sa

**Keywords:** bioremediation, *Pterocladia capillacea*, nanoparticles form, Ismate Violet 2R dye, wastewater treatments, isotherms, kinetics, pseudo-second-order, BET, FTIR, SEM

## Abstract

Based on their functional groups, the use of various seaweed forms in phytoremediation has recently gained significant eco-friendly importance. The objective of this study was to determine whether a novel, sustainable, and ecologically acceptable adsorbent could be employed to remove toxic textile dye (Ismate Violet 2R (IV2R)) from an aqueous solution. The low-cost adsorbent was prepared from the nanoparticles form of the native red seaweed species, *Pterocladia capillacea*. Before and after the adsorption procedure, comprehensive characterization experiments on the bio-adsorbent were carried out, including BET, SEM, FTIR, UV, and dynamic light scattering (DLS) examination. The adsorption performance of the prepared nano-*Pterocladia capillacea* was optimized by adjusting operating parameters such as the initial dye concentration of 60 mg L^−1^, pH of 2, and contact time of 15 min, all of which were obtained by batch experiments in the lab. At the optimum conditions, the prepared adsorbent had maximum removal effectiveness of 87.2%. Most typical kinetics and isotherm models were used to test the experimental results. The equilibrium data fit well with the Langmuir isotherm model, with comparatively higher R^2^ values and fewer standard errors, while the pseudo-second-order kinetic model fits better with a decent correlation coefficient. Thermodynamic parameters revealed that the sorption process on nano-alga was exothermic and spontaneous.

## 1. Introduction

Dyes are one of the most dangerous pollutants found in industrial effluents. Their presence in water bodies prevents light penetration, prohibits aquatic flora from photosynthesis, and causes harm to all ecosystem components, which must be dealt with [1,2,3,4,5]. Dyes are a type of organic chemical with a complex aromatic molecular structure that can give other materials a bright and deep color. Approximately 50% of all dye output in the world is wasted during the dying process and ends up in textile effluents [6]. Furthermore, in natural aquatic habitats, the presence of very low quantities of dyes in water (less than 1 ppm of some dyes) is highly visible, difficult to biodegrade, and difficult to eliminate.

Dyes are also more stable and difficult to biodegrade due to their complex aromatic molecular structures [7]. Dyes can also induce allergic reactions, dermatitis, and skin irritation in humans, as well as cancer and mutation [8,9]. As a result, removing them from industrial effluents before they are discharged into the environment is important. Over the years, a variety of technologies have been used to treat dye-containing wastewaters, such as flocculation, membrane filtration, precipitation, electrochemical, and adsorption, which have all been successful in removing different dyes and pigments from the aqueous phase [1,10]. Each method has its package of advantages and cons. The adsorption approach is the most effective method for removing synthetic dyes from aqueous effluents [11,12,13,14]. The dye species are transferred from the water effluent to a solid phase, leading to a low effluent volume. After that, the adsorbent can be regenerated or stored in a dry location away from the environment [14,15].

Aquatic plants, fungi, and bacteria represent three different types of adsorbent materials that are readily available, with the latter two potentially providing more alternatives. Waste materials or biomass by-products from large-scale fermentation activities are a convenient source of novel adsorbents [16]. Waste mycelium, for instance, is readily available in large numbers for the removal of dyes. Compared to terrestrial plants, aquatic plants (microalgae and macroalgae) have more importance in the applications of several bioindustries [17,18,19,20,21]. While aquatic organisms are rich sources of bioactive compounds higher than terrestrial organisms, aquatic plants are the most potent source of several biomaterials. They may be utilized as food, feed, fertilizers, and fine biochemicals [22,23,24,25,26]. On the other hand, aquatic plants are the main producers of oxygen on our planet. Generally, aquatic organisms have a wide range of bioactive compounds that can be positively used in bioremediation [26,27,28,29,30,31]. Seaweed (macroalgae) contains polysaccharides such as alginate, carrageenan, and fucoidan. Polysaccharides present in seaweed are one of the most common organic substances in the ocean. Today, seaweed polysaccharides are used mostly as a biological stimulant in agriculture to enhance seed germination and plant development and improve plant defense responses [32,33,34,35]. Moreover, the polysaccharides of seaweed play an important role as aquafeed additives [36,37].

The different forms of seaweeds could be a more efficient and low-cost dye uptake medium. Red seaweed *Pterocladia capillacea* is a marine red alga in which the red pigment phycoerythrin masks the chlorophyll. In general, *P. capillacea* is multicellular and usually small to moderate in size, with hollow leaves that have a cartilaginous texture. They spend their time in the Mediterranean Sea on rocks near the coast and in shallow water [2]. The liquid extract of this species previously showed significant application in the field of agriculture growth enhancer [32,33,34,35] and aquaculture feed additive [36,37]. On the other hand, the dried form of *P. capillacea* showed a positive impact in wastewater treatments [2,38].

Nanoparticles have been extensively utilized in bioremediation, due to their high specific surface area that improves the removal reaction [39]. Algal-cells nanoparticles are increasingly becoming essential in many biological applications [40,41]. Many studies reported that the nanoparticles of the red seaweed species *P. capillacea* showed antimicrobial activities [42,43], anticancer activities [44], and biomedical applications [45]. There is no information available about the potential applications of the nanoparticle form of the red seaweed species *P. capillacea* in the bioremediation capacity of toxic dyes from an aqueous solution.

By performing batch investigations, this work attempts to prepare and evaluate the possibilities of using *P. capillacea* nanoparticles for the elimination of harmful Ismate Violet 2R (IV2R) from an aqueous solution. Contact time, adsorbent dose, pH, starting concentration, temperature, and the amount of *P. capillacea* nanoparticles doped were studied. The adsorption isotherm, kinetic, and thermodynamic data approaches were used to investigate the adsorbents’ equilibrium sorption behavior. The optimal isotherm to correlate the experimental data was determined by fitting experimental data to various isotherm equations. Moreover, the adsorption process of the dye onto these adsorbents was examined using BET, SEM, FTIR, and UV analysis.

## 2. Results and Discussion

### 2.1. Adsorbent Characterization

#### 2.1.1. FTIR Spectra

**Figure 1:**
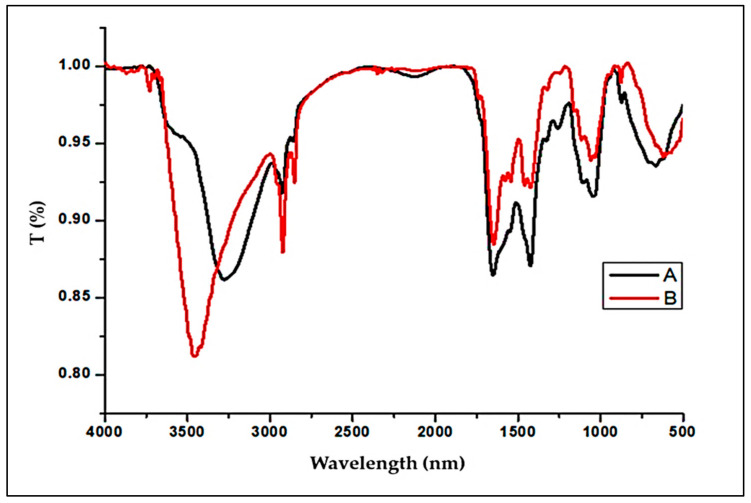
FTIR spectroscopy of *P. capillacea* nanoparticles before (A) and after adsorption (B) of IV2R dye from an aqueous solution.

The type and number of functional groups on the surface of the adsorbent have a big impact on IV2R adsorption [11]. As shown in Figure 1 and Table 1, the functional groups of the adsorbent were identified using an FTIR spectrometer. The bond –OH and –NH groups are responsible for the broadband at 3275–3720 cm^−1^. The aliphatic C–H groups were identified by the peaks at 2950–2900 cm^−1^. The peaks at 2861 and 2853 cm^−1^, respectively, are caused by asymmetric CH3 stretching and both asymmetric and symmetric CH2 stretching [12] and are seen often in *P. capillacea* nanoparticles. After remediation, the peak at 1737 cm^−1^ could be attributed to C=O of carboxylic or ester groups, while the C=O or C=C groups may be responsible for the peaks around 1650 and 1647 cm^−1^. Aromatic rings can be conjugated or non-conjugated with these groups [2], while the peak at 1101 cm^−1^ can be attributable to ether group C–O stretching. –C=O stretches aldehydes, ketones, and carboxylates could be ascribed to the adsorption peaks in the 1730–1390 cm^−1^ range [13]. Stretching vibrations –C–O, –C–C, and –C–OH were also discovered near the adsorption peaks in the 1300–1000 cm^−1^ area [14].

The stretching vibrations of –P–O, –S–O, and aromatic –CH could be responsible for the adsorption peaks in the range of 750–900 cm^−1^ [14]. The P-O-C linkages of the organic phosphate groups were assigned to the wavenumber of 1245 cm^−1^ [15]. Furthermore, a peak of red alga at 1157 cm^−1^ identifies the existence of bisulfate (HSO_4_), which is found at 871 and 878, indicating the presence of H_2_PO_4_ or PO_4_ [16]. The peaks situated at 871 and 876 cm^−1^ are typical of Silicate [2], while the signal at 576 cm^−1^ is attributed to an asymmetric deformation vibration of P=O in ${\mathrm{PO}}_{4}^{3-}$ [46]. Phosphate groups could be responsible for several bands in the fingerprint regions [47]. Furthermore, the peaks around (1041.56–1155.36 cm^−1^) and (551.64–898.8 cm^−1^) are thought to belong to the Si–O–Si and Si–H groups, according to Al-Sultani and Al-Seroury [48]. Additional peaks at 603–893 cm^−1^ can be attributed to aromatic compound bending modes.

Additionally, in this analysis, we can note the convergence of functional groups, and this could be due to the mechanism of the physical adsorption process related to the Vandervals forces, and therefore the functional groups are similar before and after adsorption. Therefore, there will be no significant change in the groups as a result of the stability on the surface due to the occurrence of adsorption on the surface. Moreover, several biomolecules, proteins, and polysaccharides have been shown to include functional groups such as carboxyl, carbonyl, hydroxyl, amino, phosphoryl, and sulphide. The various functional groups have a high affinity for IV2R dye, which allows them to form complexes with IV2R dye ions. Because most cell walls are complex, numerous potential mechanisms for dye uptake by non-living biomass have been suggested, including microprecipitation, ion exchange, and complexation [49,50]. Because untreated biomass contains light metal ions including K^+^, Na^+^, Ca^2+^, and Mg^2+^, the adsorption of copper and other metals in IV2R dye can be mostly attributed to the ion exchange with calcium. The uptake of Cu(II) in dye resulted in a considerable release of Ca^2+^, Mg^2+^, K^+^, and H+ from the sorbent [2]. This could indicate that the metals are removing these cations. When comparing *P. capillacea* nanoparticles before and after adsorption using FTIR, it was found that the formation of new absorption bands after adsorption, as well as changes in absorption intensity, shifts in the wavenumber of functional groups in the raw sample, and differences in frequency bands, could be due to the interaction of IV2R dye ions with active sites of adsorbents. IV2R dye ions were linked to the active sites of the adsorbents by electrostatic attraction or complexation.

#### 2.1.2. Scanning Electron Micrograph

**Figure 2:**
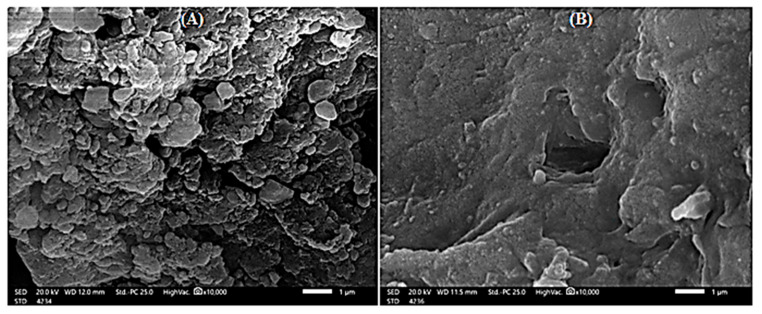
Scanning electron micrograph of *P. capillacea* nanoparticles before (**A**) and after (**B**) adsorption of IV2R dye.

The pore size and porosity of adsorbent materials have the greatest influence on the dye removal process [51,52]. In the current study, the morphological differences between the *P. capillacea* nanoparticles before and after adsorption of IV2R dye ions are investigated using SEM, as shown in Figure 2. 

Due to pores, fragments, and the irregular surface of the algae nanoparticle, the shape of this material can enhance the sorption of dye ions in different sections of this material. As a result of the shape, as well as the fact that high levels of silicate and other functional groups such as carboxyl, amino, and hydroxyl can retain cationic pollutants due to their negative net electrostatic charge, the algae was concentrated. It may be concluded that the morphological profile of this material is suitable for the adsorption of dye ions.

#### 2.1.3. UV–Vis Absorption Spectrum Properties

**Figure 3:**
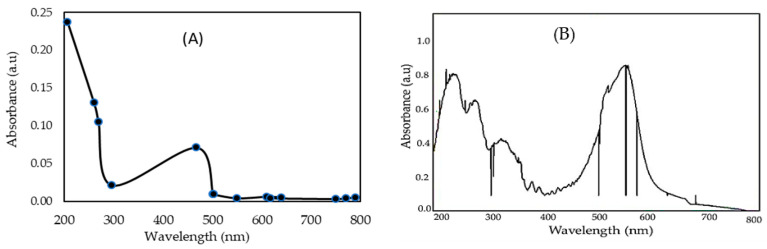
UV-Visible Spectrum of *P. capillacea* nanoparticles (**A**) and IV2R dye concentration (**B**).

The bioactive compound in the crude extract of *P. capillacea* nanoparticles was studied spectroscopically for further validation. The extracts were scanned in the wavelength range of 200–800 nm using a Shimadzu Spectrophotometer to determine the UV–Vis range of the purified sample of *P. capillacea* nanoparticles, and distinct peaks were observed (Figure 3A). Using a UV-mini 1240 UV-VIS Shimadzu spectrophotometer, the examined results were acquired from the absorbance at a 207 nm wavelength (maximum absorption peak). The absorption spectrum of the nano *P. capillacea* extract revealed a broad absorbance range between 300 and 700 nm, with a peak at 441 nm in the visible part of the solar spectrum (Figure 3A) [53]. The presence of phenolic and alkaloid chemicals in the marine algal *Sargassum wightii* is revealed by the peak occurrence at 234–676 nm [54]. The extract possesses some comparable alkaloids, flavonoids, and glycoside chemicals as described [55], based on a comparison of the spectra of seeds and flowers.

In contrast, the IV2R dye concentration (C_0_) in solution at t = 0 min was determined by monitoring the wavelength range of 200–800 nm using a Shimadzu Spectrophotometer to determine the UV–Vis range (Figure 3B). The profile showed the IV2R dye has high absorbance at 500–560 nm (Green color region) and 200–245 nm. In addition, the higher absorption of IV2R dye was obtained at a wavelength of 550 nm. The absorption bands at 256 nm are attributed to the aromatic character or chloro-triazine group C_3_H_2_ClN3 [56], while 219–230 nm may be features of the Naphthalene (C_10_H_8_) in cyclohexan [57,58]. The absorption peak at 209 and 250 nm is due to sulfanilic acid, (p-Aminobenzenesulfonic acid) C_6_H_7_NO_3_S [59].

#### 2.1.4. BET Examination

**Figure 4:**
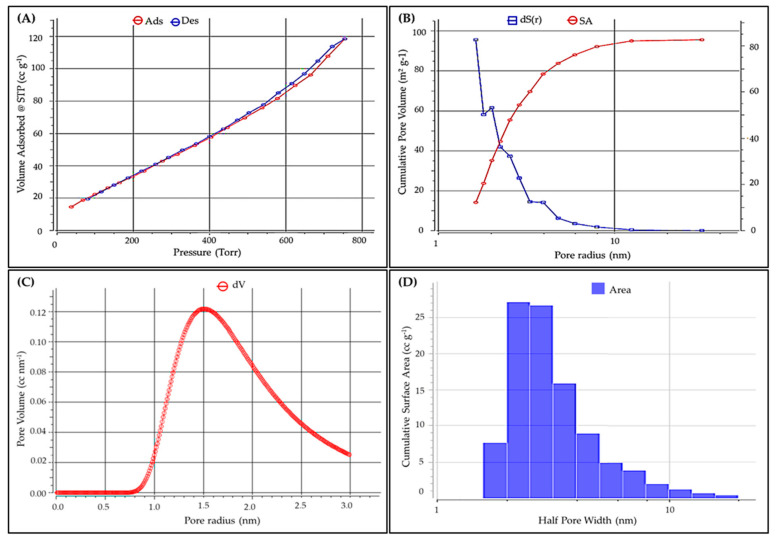
BET investigation of *P. capillacea* nanoparticle isotherm–adsorption–desorption (**A**), DH–desorption–dV(r) (**B**), DA method pore volume (**C**), and DFT method histogram surface area (**D**).

The BET analysis using physical gas adsorption of nitrogen gas at an analytical temperature of 77 K can accurately measure and quantify the specific surface area of *P. capillacea* nanoparticles, as shown in Figure 4 and Table 2. The BET-specific surface area was 128.3 m^2^ g^−1^, while the mean BJH desorption was 93.635 m^2^ g^−1^. However, a total pore volume of 0.183635 cc g^−1^ was used to explore the potential differences in nanomaterial density values. The average pore radius of *P. capillacea* was found to be 1.0628 nm. *P. capillacea* had an average pore size of 2.86258 nm.

### 2.2. Factors Influencing Batch Adsorption

Based on the batch process results, five variables were investigated to determine the optimum conditions for IV2R removal from an aqueous solution, namely solution pH, initial IV2R concentration, recovery time, adsorbent dosage, and temperature. The development of an industrial-scale dye removal treatment procedure will be substantially aided by in-depth research and the optimization of these parameters [31]. As a result, these characteristics will be discussed in the next section.

#### 2.2.1. Initial IV2R Concentrations

**Figure 5:**
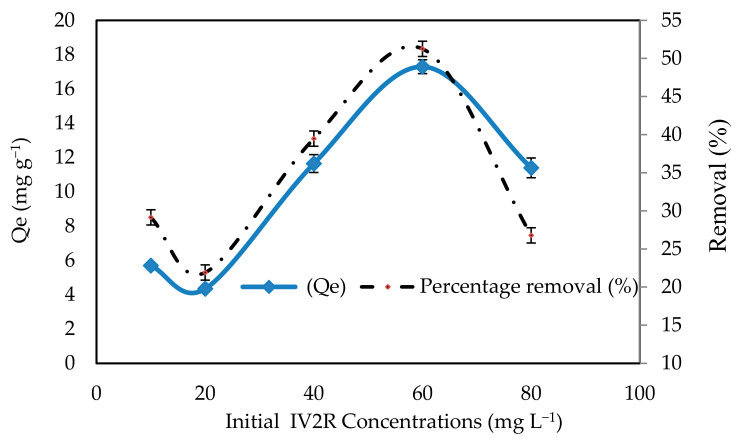
The removal efficiency for *P. capillacea* nanoparticles with several IV2R concentrations.

Different initial concentrations of IV2R had a significant impact on the adsorption process, and as the initial concentration increased, so did the adsorption capacity. The removal effectiveness of IV2R fell to 26.7% at initial doses of 10 to 60 mg L^−1^ and then reduced beyond 60 mg L^−1^ (Figure 5). The decline in dye removal percentage may be attributed to the fact that all adsorbents have a finite number of active sites that become saturated at a specific concentration [60]. In addition, because the adsorbent dose is fixed, there are a fixed number of active sites, resulting in a drop in the removal percentage as the concentration rises. Furthermore, a significantly higher percentage of sorption was seen at low initial IV2R dye concentrations [61]. However, as the initial concentration of IV2R dyes increases, the adsorption capacity (q_e_) increases. When the dye’s initial concentration was increased from 10 to 60 mg L^−1^, the adsorption capacity (q_e_) increased from 5.7 to 17.3 mg g^−1^. This is due to the interaction between the dye molecules and the surface of the adsorbent, which is enhanced when the initial concentration of the dye is increased [62]. So, when the initial dye concentration is high, the number of ions competing for the existing sites on the external adsorbent is excessive and high, therefore, resulting in greater IV2R sorption capacity. However, in the range of 10 to 20 mg L^−1^, the adsorption capacity (q_e_) decreased, which may be caused by the saturation of sorption sites on the surface of *P. capillacea* nanoparticles. Similarly, the lower uptake at higher concentrations resulted from an increased ratio of the initial adsorption number of moles of the dye to the available surface area; hence, the fraction becomes dependent on the initial concentration [63]. In general, the increase in the initial concentration of the dye enhances the interaction between the dye molecules and the surface of the adsorbent [64].

Kareem et al. [65] explained that a large number of vacant surface sites were available for adsorption during the initial stage. However, with a lapse in adsorption time, the remaining vacant surface sites were difficult to occupy due to the steric barrier between the IV2R dye adsorbed on the surface of the adsorbent and the solution phase.

#### 2.2.2. Initial Adsorbent (*P. Capillacea* Nanoparticles) Doses

**Figure 6:**
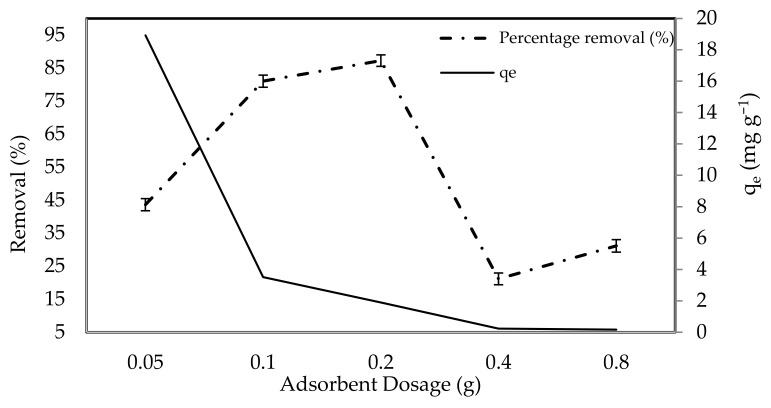
Influence of adsorbent dose on IV2R adsorption.

The experiments were carried out by adjusting the adsorbent dose (nanoparticles) from 0.05 to 0.8 g at a fixed initial dye concentration of 10 mg L^−1^, pH = 3, a contact period of 120 min, and a shaking speed of 120 rpm, to determine the effects of adsorbent dosage on IV2R dye removal efficiency (Figure 6). When *P. capillacea* nanoparticles increased from 0.05 to 0.2 g, the adsorption capacity improved from 43.6% to 87.2%, while the greatest adsorption capacity was attained at 0.05 g with 18.9 mg g^−1^. This trend could be explained by the fact that increasing the adsorbent dosage increases the surface area, resulting in more active sites available to bind dye from the aqueous phase [66]. Due to the obvious aggregation and overlapping of adsorbent nanoparticles in the solution at masses greater than 0.2 g, the amount of IV2R was reduced, resulting in a decrease in the surface area for dye uptake. The removal efficiency increased when (*P. capillacea* nanoparticles) doses increased from 0.4 to 0.8 g. This is possibly owing to the decrease in the removal efficiency by increasing the adsorbent mass due to the overlapping and aggregation of adsorption sites decreasing the surface area available to pollutant ions, as well as the resistance to mass transfer of IV2R dye from the bulk liquid of the solution to the solid surface, which is significantly converted at high adsorbent loading [67]. Likewise, Khattri and Singh reported that the primary influence explaining these properties is that sorption sites remain unsaturated during the sorption reaction while the number of sites accessible for adsorption sites is raised by increasing the sorbent dosage [68]. This result is similar to a finding of Ai et al. [69] and Lee et al. [64]. As a result, for the next tests, an adsorbent mass of 0.1 g was used.

#### 2.2.3. Initial Temperature

**Figure 7:**
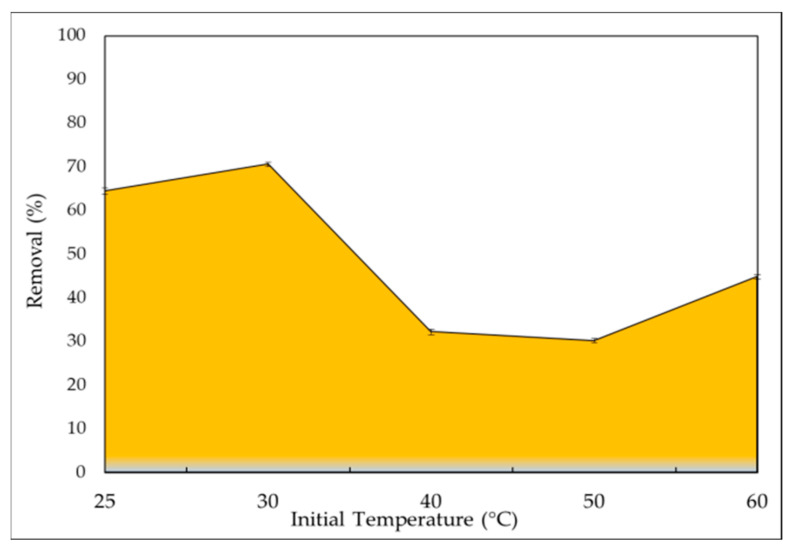
IV2R uptake capacities at varying temperature intervals.

When temperature increases, it usually improves the adsorptive removal of adsorptive pollutants by increasing the surface activity and kinetic energy of the adsorbate, but it can also harm the physical structure of the adsorbent [70]. Within a temperature range of 25 to 60 °C, the influence of temperature on the removal efficiency was examined, as presented in Figure 7. The maximum removal percentage (70.5%) of the IV2R was observed at 30 °C. Several studies have found that if the temperature rises above 30 °C, the removal percentages decrease. This could be due to an increase in the relative desorption of IV2R from the solid to the liquid phase, the deactivation of the adsorbent surface, the destruction of active sites on the adsorbent surface due to bond disruption [71], or the weakening of the sorbent active site binding forces and the sorbate species, as well as between adjacent molecules of the sorbed phase [72]. As shown in Figure 7, increasing the temperature from 25 to 30 °C has a greater effect on the adsorption process, allowing studies to be conducted at room temperature without any modifications. This suggests that the adsorption process is exothermic when the temperature was reduced from 40 to 60 °C. These findings also suggest that the adsorption process is physisorption. In this situation, the adsorptive forces between the dye molecules and the active sites on the adsorbent surface may be reduced by increasing the temperature [73].

#### 2.2.4. Initial Contact Time

**Figure 8:**
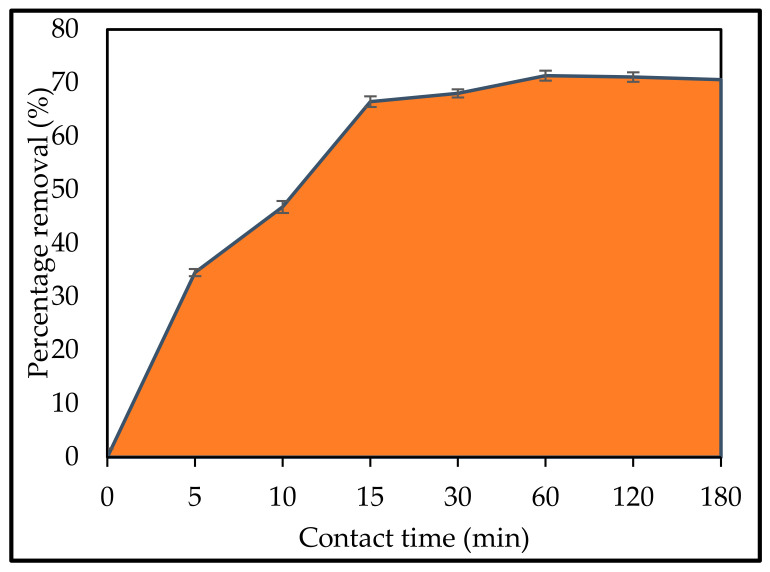
Effect of contact time intervals in IV2R removal.

It was critical to determine how long it would take to reach equilibrium adsorption. At an initial concentration of 10 mg L^−1^ and pH = 2, the influence of contact time on the adsorption process was examined. A rotary shaker was used to agitate the solution at a speed of 120 rpm. The results of the adsorption efficiency vs. contact time for IV2R dye solutions are shown in Figure 8. 

The removal of more than 63.9 and 68% of IV2R concentration occurred in the first 15 and 30 min, respectively, and thus the rate of *P. capillacea* nanoparticle adsorption was observed to be slow after that. The availability of the positively charged surface of the adsorbent for adsorption of anionic IV2R in the solution at pH 2 explains the rapid adsorption at the initial contact time. The electrostatic hindrance or repulsion between the adsorbed negatively charged sorbate species onto the surface of *P. capillacea* nanoparticles and the available anionic sorbate species in the solution, as well as the slow pore diffusion of the solute ion into the bulk of the adsorbent, are likely responsible for the later slow rate of IV2R adsorption [74,75,76]. The equilibrium was found to be nearly 60 min when the maximum IV2R adsorption onto *P. capillacea* nanoparticles was reached.

#### 2.2.5. Initial pH

**Figure 9:**
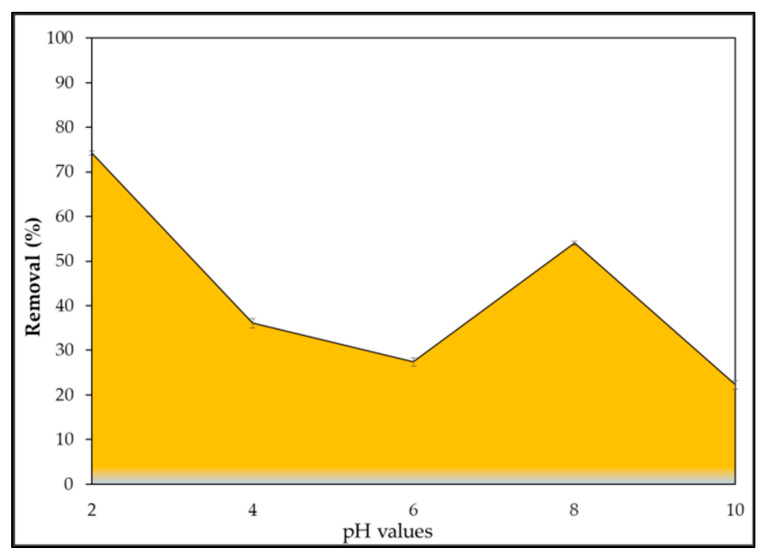
Effect of several pH values on IV2R removal.

The pH of the solution significantly affects the number of dye ions adsorbed onto adsorbents as it affects the properties of the adsorbents, in addition to the speciation of dye ions in an aqueous solution [77]. Furthermore, the pH solution appears to influence the solution chemistry of dyes as well as the activity of the functional groups of the biomass and hence plays a major role in adsorption [31]. According to the current findings, the largest removal of IV2R occurred at pH 2 and the highest IV2R removal of 74.1%, where the adsorption capacity was 19.59 mg g^−1^, and subsequently declined as pH increased, reaching 22% at pH 10 (Figure 9). The nature of *P. capillacea* nanoparticles’ adsorption at different pH, the type of ionic state of the functional group of the sorbent, and the dye chemistry can all explain this behavior [78]. As can be seen in the figure, the lowest uptake value was recorded at pH 8 and the maximum uptake value was observed at pH 2, with uptake values decreasing dramatically as the pH increased. Furthermore, at the very low pH of 2, the surface of the sorbent would be surrounded by hydronium ions, which would increase the attractive interactions between IV2R dye ions and binding sites of *P. capillacea* nanoparticles [79]. Furthermore, functional groups such as carbonyl and hydroxyl on the adsorbent surface can polarize, leading to electrostatic interaction in addition to hydrogen bonding and van der Walls interaction. 

Moreover, the electrostatic attraction between negatively charged dye anions and positively charged cell surfaces may explain why it so favors the adsorption of the anions at lower pH values. The positive charge on the solution interface increases at low pH, and the adsorbent surface appears positively charged, resulting in decreased cationic dye adsorption and increased anionic dye adsorption [80]. As the pH increased, the overall surface charge on *P. capillacea* nanoparticles became negative and the adsorption of IV2R dye decreased [81]. Within the pH range of 6–8, the drop in adsorption (percentage elimination of approximately 53.96%) could be caused by the drop in the polarization of the outer surface of *P. capillacea* nanoparticles [82]. The lower adsorption of IV2R, an anionic dye, at an alkaline pH is provable due to the presence of excess OH^-^ ions competing with the dye anions for the adsorption sites. Furthermore, the decrease in IV2R adsorption with an increasing pH is owing to competition between anionic dye and excess OH- ions in the solution, which may be attributed to the fact that OH- ions with high concentrations and mobility prefer adsorbed compared to dye anions [83]. Due to electrostatic repulsion, a negatively charged surface site on *P. capillacea* nanoparticles does not favor the adsorption of anionic IV2R molecules. That could be due to the developed adsorbent hydrophobic characteristic, which caused it to absorb hydrogen onto its surface when submerged in water, making it positively charged [84].

### 2.3. Equilibrium Studies

**Figure 10:**
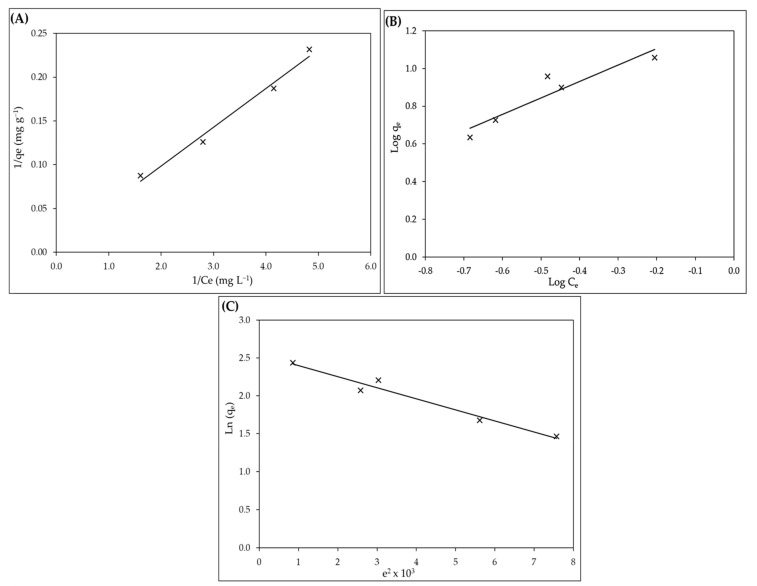
Langmuir (**A**), Freundlich (**B**), and Dubinin–Radushkevich (**C**) plot for IV2R dye adsorption.

The relationship between the amount of adsorbate taken up by the adsorbent and the adsorbate concentration remaining in the solution is described by an adsorption isotherm. To assess experimental adsorption equilibrium data, there are numerous formulae. The adsorption mechanism, surface characteristics, and affinity of the adsorbent are generally revealed by the equation variables of these equilibrium models. The Langmuir [85], Freundlich [86], and Dubinin–Radushkevic [87] isotherm models were investigated in this study. The isotherms of adsorption of IV2R on *P. capillacea* nanoparticles were carried out using the best experimental conditions previously described (Table 3 and Figure 10A–C). The Langmuir model (Figure 10A), which is widely used in adsorption science, assumes that solute adsorption is limited to monolayer sites inside the adsorbent and that there is no interaction between adsorbate molecules. The results showed that *P. capillacea* nanoparticles ingest IV2R from an aqueous solution in an acceptable manner. Furthermore, the greatest quantity of IV2R absorption for *P. capillacea* nanoparticles was 100 mg g^−1^, with b being the model adsorption constant, as shown in Table 3 and Figure 10A. The Freundlich model (Figure 10B) assumes that absorption occurs on the adsorbent’s heterogeneous surfaces and that the adsorbate particles may interact. The adsorption capacity and adsorption intensity are represented by the Freundlich constants K_f_ and n, respectively. The constants’ values are found by graphing log q_e_ versus log C_e_, as shown in Table 3 and Figure 10B. With a smaller correlation coefficient, the Freundlich isotherm does not fit the experimental data well. 

The values of the Freundlich constant found, n > 1, indicated that the adsorption process is favorable. When IV2R adsorbs on *P. capillacea* nanoparticles, K_f_ was increased. For IV2R, the values of Freundlich’s constant n range from 1 to 10, showing that the chemical interactions produced between the adsorbents and IV2R are strong, which could be attributed to the pollutant’s reactive nature. The Dubinin–Radushkevich (D-R) isotherm model was applied to the equilibrium data to assess whether the adsorption process was physical or chemical [78]. When IV2R is removed from *P. capillacea* nanoparticles, the E parameter of the D-R isotherm model is lower, indicating that the ion exchange process is slower. Physical processes have values below 8 kJ mol^−1^, whereas chemical processes have values between 8 and 16 kJ mol^−1^ (Table 3 and Figure 10C).

Physical interactions involving functional groups and electron transfer between the adsorbate and adsorbents resulted in the removal of IV2R on the adsorbent, as shown in Table 3. Both Langmuir and D-R isotherm models have fit the experimental results well, indicating that IV2R monolayer coverage occurs on the surface of *P. capillacea* nanoparticles.

### 2.4. Error Functions Studies

As shown in Table 4, the Langmuir model had reduced errors in virtually all of the examples, while the Freundlich and Dubinin–Radushkevich models did not exhibit great accuracy. The values of the error functions are provided in Table 4. The Langmuir isotherm model can be more relevant in this situation for understanding the adsorption process of IV2R ions by *P. capillacea* nanoparticles. The only way to determine the best isotherm model is to examine the correlation coefficient (R^2^). This indicator is limited to fitting isotherm models with linear forms [88] despite its efficiency.

### 2.5. Kinetic Studies

**Figure 11:**
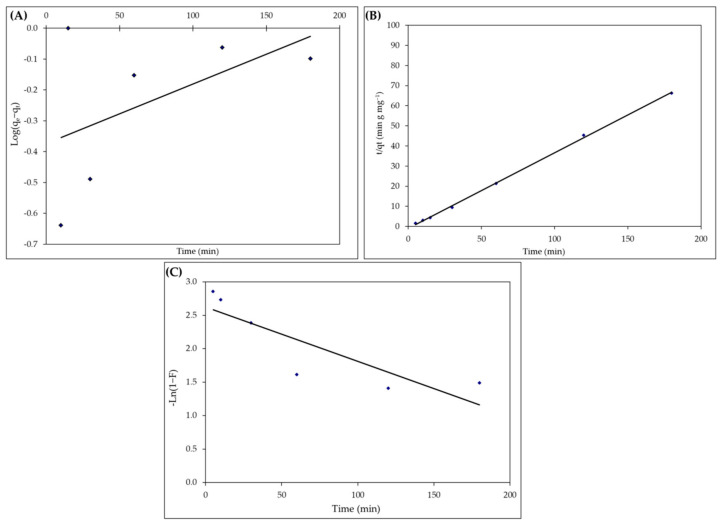
Pseudo-first-order (**A**), pseudo-second-order (**B**), and film-diffusion-kinetic (**C**) kinetics models for IV2R sorption.

Pseudo-first-order, pseudo-second-order, and film-diffusion-kinetic models were performed to describe the experimental data for analyzing the sorption kinetics of IV2R (Table 5 and Figure 11A–C). According to the Lagergren pseudo-first-order model, the rate of change in solute uptake is related to the difference in the saturation concentration and the amount of solid uptake over time, implying that the rate of adsorption site occupation is proportional to the number of vacant sites. This equation has the following general form (1):

dqt/dt = K_1_ (q_e_ − q_t_) (1)

where q_e_ represents the quantity of IV2R adsorbed at equilibrium (mg g^−1^), q_t_ represents the amount of IV2R adsorbed at any time t (mg g^−1^), and K_1_ represents the adsorption rate constant (1/min). When certain boundary conditions were applied, then: q_t_ = 0 at t = 0; q_t_ = q_t_ at t = t and integrating Equation (1), the following equation [89] may be obtained (2):

Log (q_e_− q_t_) = Log q_e_ − (K_1_/2.303) t (2)

Table 5 shows the rate constant (K_1_) and the equilibrium amount of IV2R (q_e_) calculated from the slope and intercept of the Log (q_e_ − q_t_) against the time (t) plot.

According to Ho et al. [90], the general Equation (3) of pseudo-second-order kinetics is:

d q_t_/dt = K_2_(q_e_ − q_t_)^2^ (3)

The pseudo-second-order equilibrium rate constant (g mg^−1^ min) is denoted by K_2_. After integrating, Equation (4) can be linearized by using boundary conditions (q_t_ = 0 at t = 0 and q_t_ = q_t_ at t = t).

Ln (1 − F) = K_FD_ (t) (4)

where F = q_e_/q_t_ is called the fractional attainment of the equilibrium and K_FD_ is the film diffusion constant rate.

As shown in Figure 11A, the coefficient of determination (R^2^) for the pseudo-first-order kinetic model was low. As an alternative, the pseudo-second-order equation, which describes the solid phase’s sorption equilibrium capacity, is commonly used in kinetics research. In Figure 11B, the linear plot of t/q_t_ against t yielded a straight line.

Table 5 shows the pseudo-second-order rate parameters, K_2_, as well as the coefficient of determination, R^2^. The result shows that K_2_ values were high, indicating that a higher dose benefits the adsorption process by boosting the adsorption rate and capacity. Furthermore, the q_e_ calc. and q_e_ (Exp.) have only a minor difference. 

Boyd, et al. [91] proposed his film-diffusion-kinetic model to describe the adsorption of ions from aqueous solutions to solid-phase diffusion and suggested the film diffusion equation as follows. We obtained a linear relationship by plotting Ln(1 − F) versus time, which did not pass the origin, indicating that film diffusion is not the rate-determining step in the whole adsorption process. This was confirmed by the absorption correlation coefficient, which is low (0.228), as shown in Figure 11C and Table 5. The data already showed a satisfactory correlation with the pseudo-second-order model, with a coefficient of determination of 0.999 for the linear plots.

### 2.6. Adsorption of Thermodynamics

Table 6 shows the estimated thermodynamic characteristics for the adsorption system, such as standard enthalpy change ∆H^O^, standard entropy change (DS^O^) ∆S^O^, and Standard free energy changes ∆G^O^. The adsorption of IV2R ions has indeed been found to decrease as the temperature rises from 30 to 60 °C. Table 6 reveals that the ∆H^O^ values obtained have been negative and that the distribution coefficient values, Kd, declined as the temperature increased, indicating that the adsorption is exothermic. Gibbs free energy is also useful in determining the type of adsorption, such as physical or chemical. ∆G^O^ up to −20 kJ mol^−1^ has been reported to be consistent with the electrostatic interaction between the adsorbent surface and dye ions (physical adsorption). Meanwhile, ∆G^O^ values less than −40 kJ mol^−1^ involve charge information exchange or transfer from the biomass surface to the dye ion to form a coordinate bond (chemical adsorption) [92]. ∆G^O^ values higher than −40 kJ mol^−1^ involve charge sharing or transfer from the biomass surface to the dye ion to form a coordinate bond (chemical adsorption). The ∆G^O^ values for the IV2R adsorption formed in this work are −10 kJ mol^−1^, indicating that physical adsorption via a physical process that involves weak forces of attraction was the dominating mechanism. The growing unpredictability at the solid/liquid interface during the adsorption of IV2R ions on *P. capillacea* nanoparticles was suggested by the positive value of ∆S^O^. The negative values of ∆G^O^ suggested spontaneous adsorption, and the reduction in ∆G^O^ value with a rising temperature indicated that the reaction was favorable at lower temperatures.

### 2.7. Proposed Adsorption Mechanism

Most red algae are filamentous and multicellular. Most red algal cell walls have a hard inner portion made up of microfibrils and a mucilaginous matrix. The matrix is made up of sulfated galactose polymers named agar, funori, porphysan, and carrageenan, which give the red algae their flexible, slippery texture.

The main observations that confirm this mechanism are as follows:

The presence of various functional groups on the surface of *P. capillacea* nanoparticles, such as hydroxyl, carboxyl, amine groups, and others, is strongly dependent on the adsorption of IV2R dye from an aqueous solution by nanoparticles derived from the marine red alga, as shown in the FTIR and UV spectral results in Figure 1 and Figure 3.

Upon protonation and deprotonation, the surface of *P. capillacea* nanoparticles’ functional groups can become charged (negative and positive) or neutral. The numerous interactions, such as electrostatic attractions, hydrogen bonding interactions, and interactions between the adsorbent and adsorbate, can be attributed to the probable adsorption mechanisms between the surface functional groups of *P. capillacea* adsorbent and IV2R. El-sikaily et al. [2] reported a similar assessment for the adsorption of pollutants on *P. capillacea*.

Furthermore, Figure 5 shows that IV2R has good adsorption in all pH ranges (2–10). This could be attributed to the formation of surface hydrogen bonds between the surface hydrogen bonds of the hydroxyl group on the *P. capillacea* nanoparticles’ surface and the nitrogen atoms and sulphonyl group of IV2R dye. Moreover, the sorption rate is very fast in such a way that the equilibrium is reached in less than 15 min.

Additionally, the ΔG^O^ values from the adsorption of thermodynamics are −10 kJ mol^−1^, indicating that the physical adsorption through a physical process involves weak forces of attraction as the dominating mechanism. Besides, the equilibrium data fitted very well to the Langmuir model, which shows favorable monolayer adsorption onto a surface containing a finite number of identical sites.

### 2.8. Comparison with Other Adsorbents

Table 7 compares the adsorption capabilities (q_max_) of several seaweed adsorbents for several pollutants. The current study concluded that among seaweed species, the nanoparticles of red seaweed *P. capillacea* have significant potential for use in the adsorptive removal of color from IV2R-laden wastewater due to its low cost, reusability, and high adsorption capability.

## 3. Conclusions

Nanoparticles of the red seaweed species, *Pterocladia capillacea,* have been used to remove the toxic IV2R dye from an aqueous solution. Before and after IV2R removal, the nanoparticles were characterized using BET, SEM, FTIR, and UV. The maximum percentage of MB removal (87.2%) was achieved at a nanoparticles biomass dosage of 0.2 g, pH of 2, 60 mg L^−1^, and 30 °C with a 30 min equilibrium time, which were shown to be the optimum adsorption conditions. The adsorption ability of *P. capillacea* nanoparticles for the IV2R dye increased with an increasing initial IV2R concentration and decreasing temperature degree. The Freundlich and Langmuir isotherm models were used to match the adsorption data. *P. capillacea* nanoparticles were tested for their adsorption capability and affinity. The Langmuir isotherm model predicted the maximum adsorption capacity (q_max_) for IV2R dye to be just 22.22 mg g^−1^. According to the results data, the experimental data were in accordance with the Langmuir isotherm (R^2^ = 0.983) and the second-order kinetic (R^2^ = 0.999).

Likewise, thermodynamic characteristics for the adsorption system, such as standard enthalpy change ∆H^O^, standard entropy change ∆S^O^, and standard free energy changes ∆G^O^, were calculated by adsorption equilibrium constants. The ∆G^O^ values for the IV2R complexes formed in this work are −10 kJ mol^−1^, indicating the physical adsorption. All ∆G^O^ values were negative; the ∆H^O^ values and ∆S^O^ values of adsorbents were −27.82 (kJ mol^−1^) and 0.117 (J mol^−1^ K^−1^), respectively. The interaction of IV2R ions with the surface of the adsorbent resulted in the development of distinct aggregates. The dye ions were attracted to the active sites of the adsorbents by electrostatic attraction or by complexation. Low cost, environmental friendliness, high uptake capacity, and non-toxicity are only a few of the benefits of this natural adsorbent. As a result, it can be used to remove IV2R dye from aqueous solutions as an effective adsorbent. This biomass can be used to provide a solution for the removal of different pollutants.

## 4. Materials and Methods

### 4.1. Adsorbent Preparation

**Figure 12:**
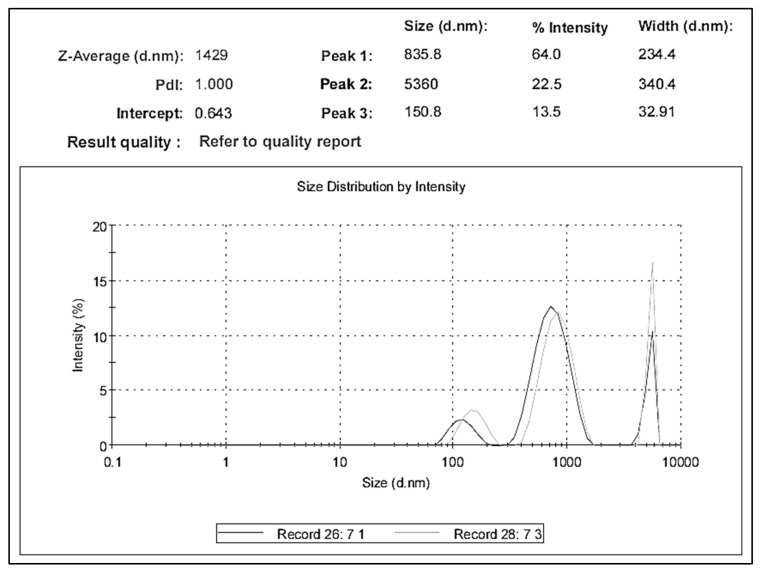
Dynamic Light Scattering (DLS) of *P. capillacea* nanoparticle size.

Red seaweed species (*P. capillacea* S.G. Gmelin) were harvested, cleaned, washed, and air-dried in shadow, powdered, and stored as described by Mansour et al. [31]. *P. capillacea* nanoparticles were prepared using ball mailing (Planetary Ball Milling Machine PM 400, Berlin, Germany). Zetasizer Nano Series HT (Malvern Analytical Ltd., Worcestershire, UK) was applied to determine the Dynamic Light Scattering (DLS) of the *P. capillacea* nanoparticle size, which showed average sizes of 150.8 nm and 835.8 nm (13.5% and 64.0%, respectively), as shown in Figure 12.

### 4.2. Dye Preparation

**Figure 13:**
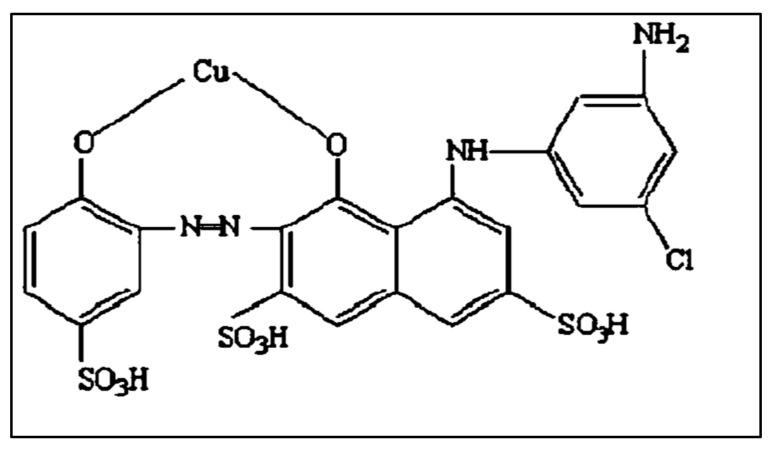
Structure of ismate violet 2R.

All solutions were prepared using de-ionized water throughout the experiments. Figure 13 shows the toxic textile dye, Ismate Violet 2R (IV2R), (C_22_H_14_N_4_O_11_S3 CuCl, Mol. wt. 700, max = 550 nm). It was utilized without being purified any further. Three sulfonate groups and another amino group are present in the dye [9]. The IV2R was dissolved in distilled water to make the stock solution. Diluting the IV2R stock solution to the desired concentrations yielded working solutions. 

### 4.3. Batch Adsorption Experiments

The adsorption studies were performed in batch mode, with 50 mL of water suspension containing 0.1 g of *P. capillacea* nanoparticles and a known concentration of the IV2R dye solution. The resulting solution was poured into a 100 mL polypropylene flask, sealed, and stirred at 120 rpm with a rotary shaker. The pH of the adsorbate and adsorbent solution was adjusted to the appropriate initial pH (from 2 to 12) using 0.1 mole hydrochloric acid or sodium hydroxide solutions. A Schott Lab 850 set pH was used to determine the pH of the liquids. Samples were taken at five-minute intervals (5, 10, 15, 30, 60, 120, and 180 min) and IV2R dye concentrations were measured. To estimate the parameters of isotherm models and thermodynamic equations, equilibrium tests were carried out at 298 K, with the flasks and shaker placed in a temperature-controlled incubator and five different initial IV2R dye concentrations (10, 20, 40, 60, and 80 mg L^−1^). Using a UV–Vis spectrophotometer with a maximum wavelength of 550 nm, the absorbance of residual supernatants solutions was determined. The amount of IV2R adsorbed per unit weight of *P. capillacea* nanoparticles at equilibrium, q_e_ (mg g^−1^), and the percentage of dye removal (R %) were calculated by the following Equations [97,98]:

q_e_ (mg g^−1^) = [(C_i_ − C_f_) × V]/W (5)

Percentage removal (%) = [(C_i_ − C_f_)/C_i_] × 100 (6)

where C_i_ and C_f_ (mg L^−1^) represent the initial primary and final IV2R concentrations at a given time, respectively; V denotes the dye mixture volume (L) and W denotes the weight of the dry adsorbent (g). Concentrations of the dye were measured at the wavelength of its maximum absorbance (λmax), which was determined by a UV–Vis spectrophotometer. The final dye concentration was determined spectrometrically corresponding to λ_max_ of the dye using the Beer–Lambert equation:

Absorbance = εCS l(7)

where l is the thickness of the absorbing medium (1 cm), CS is the concentration of the sample, and ε is the molar absorptivity.

### 4.4. Characterization of Adsorbent Material

#### 4.4.1. Fourier Transform Infrared Spectroscopy (FTIR)

The KBr disc mode was used in Fourier transform infrared spectroscopy (FTIR) by (TENSOR Series FT-IR Spectrophotometer, Borken, Germany). To obtain the spectra, the material was combined with 1% KBr and crushed into pellets, then examined in the mid-infrared region (500–4000 cm^−1^) at a 4 cm^−1^ resolution [97,98].

#### 4.4.2. Scanning Electron Microscopy (SEM)

Under a 30 kV accelerating voltage, the surface morphology of the samples was studied using a scanning electron microscope (SEM, FEI, Quanta 200, Thermo Scientific, Waltham, MA, USA). The morphological variations between the nano *A. platensis* biomass before and after IV2R ion adsorption are verified by SEM [97,98].

#### 4.4.3. Brunauer Emmett Teller (BET)

The total surface area, total pore volume, and mean pore diameter of the adsorbent were investigated. Using inert gases and a pressure atmosphere, the adsorption–desorption isotherm and pore size distribution were displayed. The NLDFT Ads. model was used to do the BET analysis. Using a 13.3 Pa pressure transducer, the analysis was carried out at 33.5 atm using inert nitrogen at 77 K and gas at 87 K [97,98].

#### 4.4.4. Ultraviolet Spectra (UV)

To evaluate the absorption spectrum (UV–vis spectrophotometer, UV-2550, Shimadzu, OSLO, Kyoto, Japan) of *P. capillacea* nanoparticles extract with a range of 200–800 nm, the nanoparticles powder was dissolved in deionized water, and diluted in 100 mL volumetric flasks [31].

### 4.5. Statistical Evaluation of the Isotherm Parameters

The IV2R ions’ equilibrium sorption was achieved by intermittently contacting 0.1 g of *P. capillacea* nanoparticles at concentrations ranging from 10 to 80 mg L^−1^ in 50 cm^3^ Pyrex conical flasks on the orbital shaker for 180 min. The combination was filtered, and the filtrate was tested with a spectrophotometer for IV2R ions concentration. The data were fitted into the Langmuir [85], Freundlich [86], and Dubinin–Radushkevic [87] isotherms, which were represented by Equations of (8), (9), and (10), respectively, as the following:

q_e_ = q_max_bC_e_/(1 + bC_e_) (8)

where C_e_ (mg L^−1^) is the adsorbate equilibrium concentration. At equilibrium, q_e_ is the amount of adsorbed IV2R per adsorbent (mg g^−1^), q_max_ is the maximal monolayer coverage capacity (mg g^−1^), and b is the Langmuir isotherm constant (L mg^−1^).

Log q_e_ = Log K_f_ + 1/n Log C_e_ (9)

where K_f_ is an approximate indicator of adsorption capacity, while 1/n is a function of the strength of adsorption in the adsorption process.

Ln q_e_ = Ln q_m_ − K ε^2^ ε = RT Ln (1 + 1/C_e_) (10)

$$E=1/\sqrt{\left(2K\right)}$$

where q_m_ (mg g^−1^) is a constant related to the adsorption capacity as well as the Polanyi potential. K (mol^2^ kJ^−2^) is the adsorption mean free energy constant; E (kJ mol^−1^) is the sorption energy constant; R (J mol^−1^ K^−1^) is the gas constant; and T (K) is the absolute temperature.

### 4.6. Error Analysis

The standard errors for each variable have been used to calculate the goodness-of-fit, correlation coefficients (R^2^), as well as error analyses (Hybrid fractional error (HYBRID), the sum of squares of errors (ERRSQ), or the root mean square errors (RMS)) between experimental and predicted data. As a result, three alternative error functions were used in this study to find the isotherm model that best represented the experimental data.

### 4.7. Hybrid Fractional Error (HYBRID)

Because it adjusts for low concentrations by balancing the absolute deviation against the fractional error and is more dependable than other error functions, the hybrid fractional error function is used. Equation (11) gives the hybrid error according to the literature [99,100,101]:

(11) $$\mathrm{HYBRID}=\frac{100}{N-P}\sum \left|\frac{q_{e,\exp}-q_{e,calc}}{q_{e,\exp}}\right|$$

where q_m_ is the isotherm model constant, q_e_ is the equilibrium capacity derived from experimental data, N is the number of data points, P is the number of isotherm parameters, and q is the average of q_e_.

### 4.8. The Root Mean Square Errors (RMS)

The following Equation (12) gives the root mean square errors (RMS) [10]:

(12) $$\mathrm{RMS}=100\times \sqrt{\frac{1}{N}\sum_{i=1}^{N}(1-\frac{q_{e,calc}}{q_{e,isotherm}}{)}^{2}}$$

### 4.9. Sum of the Squares of The Errors (ERRSQ)

The following Equation (13) gives the sum of the squares of the errors (ERRSQ) [10]:

(13) $$\mathrm{ERRSQ}=\sum_{i=1}^{N}{\left(q_{e,calc}-q_{e,isotherm}\right)}_{i}^{2}$$

### 4.10. Adsorption Thermodynamics

The Gibbs free energy change, ΔG^O^, is an important principle of non-spontaneity, and a thermodynamic analysis is required to determine if the nature of sorption techniques is spontaneous or not. The following nonlinear forms should be used to calculate the Gibbs free energy for sorption techniques at various temperatures (e.g., 25, 35, 45, and 55 °C) utilizing changes in the enthalpy ΔH° and entropy ΔS^O^ parameters [102,103,104]:

K_d_ = q_e_/C_e_ (14)

ΔG^O^ = −RT Ln K_d_ (15)

ΔG^O^ = ΔH^O^ − T ΔS^O^, or (16)

ΔG^O^ = T (ΔS^O^) + ΔH^O^ (17)

In addition to ΔG^O^, which may be obtained using Equation (16) or (17), the values of ΔH^O^ and ΔS^O^ were calculated from the intercept and slope of the methodical T vs. ΔG^O^ according to Equation (17). The apparent equilibrium constant (K_d_) (L g^−1^) of the adsorption is defined according to Potgieter et al. [101] where R is the universal gas constant (8.314 J mol^-1^ K) and T is the absolute temperature in K.

## Figures and Tables

**Table 1:** Frequency and annotations of FTIR spectroscopy analyses of *P. capillacea* nanoparticles before and after adsorption of IV2R dye.

Before Adsorption		After Adsorption		
Wave number (cm^−1^)	Annotations	Wave number (cm^−1^)	Difference (cm^−1^)	Annotations
3275.778	O–H group	3729.0164	453.2384	O–H group and –NH groups
---		3449.8535	new peaks	
2927.232	CH_2_ group	2922.8509	4.3811	Aliphatic C–H group
2861.518	---	2853.2895	8.2285	Asymmetric CH_3_ & symmetric CH_2_ stretching
2121.417	C≡C stretching	Disappearance		
		1737.5394	new peaks	C=O of the carboxylic groups or ester groups
1650.681		1647.638	3.043	C=O or C=C groups
--		1577.2542	new peaks	–C=O stretches aldehydes, ketones, and carboxylate
--		1543.0415	new peaks	C=C stretching
--		1459.093	new peaks	C–C; C–O; C=O
1424.594	–C=O stretches	1422.7408	34.499	C–C and C–O
1328.247	C–O	1323.1354	5.1116	Acyl C–O or phenol C–O
1259.681	–O–C links of the organic phosphate groups	1245.0011	14.6799	-O-C links of the organic phosphate groups
----		1157.0926	new peaks	–C–O, –C–C, and –C–OH stretching
1101.43	C–O stretching of ether groups	1113.9232	12.4932	C-O of carboxylic acids and alcohols
1044.796		1057.873	13.077	Si–O–Si and Si–H groups
		1035.3161	new peaks	Alkoxy C–O
871.9458	–P–O, –S–O, and aromatic –CH stretching or Silicate	876.6764	4.730	Silicate& bending modes of aromatic compounds & C=O stretching vibration
666.8687	C–H bend	618.765	48.10	alkene sp2 C–H bend
		576.087	new peaks	$P=O\;\mathrm{in}\;{\mathrm{PO}}_{4}^{3-}$

**Table 2:** Main properties of powdered *P. capillacea* nanoparticles.

Parameters	Value
Polarizability	1.46 (mL mol^−1^) × 1 × 10^−24^
Average Pore Size	2.86258 nm
Average Particle radius	1.0628 nm
Total Pore Volume	0.183635 cc g^−1^
Multipoint BET	128.3 m^2^ g^−1^
Langmuir method	210.311 m^2^ g^−1^
BJH adsorption	95.7161 m^2^ g^−1^
BJH desorption	93.635 m^2^ g^−1^
DH adsorption	98.1558 m^2^ g^−1^
DH desorption	95.5924 m^2^ g^−1^
V-t method micropore surface area	18.3904 m^2^ g^−1^
V-t method external surface area	109.91 m^2^ g^−1^
Molecular Density	6.7 (mol cm^2^)

**Table 3:** Sorption isotherm parameters for the removal of IV2R onto *P. capillacea* nanoparticles.

Model	Parameters	Value
Langmuir	Q_m_ (mg g^−1^)	100
	b	0.226
	R^2^	0.983
Freundlich	n	1.145
	1/n	0.873
	K_f_ (mg^1−1/n^ L^1/n^ g^−1^)	15.84
	R^2^	0.879
Dubinin-Radushkevich	Q_m_ (mol kg^−1^)	12.77
	K (mol kJ^−1^)^2^	2.55
	E (kJ mol^−1^)	0.113
	R^2^	0.962

**Table 4:** Values of three different error analyses of isotherm models for the adsorption of IV2R by *P. capillacea* nanoparticle.

Error Analyses	Langmuir	Freundlich	Dubinin-Radushkevich
Hybrid	565.270	1418.35	2159.503
ERRSQ	1556.645	7675.95	10,116.713
RMS	13.181	15.29	19.752

**Table 5:** Adsorption kinetics parameters.

Model	Parameters			
1st-order-kinetic model	R^2^	K_1_ × 10^3^ (1 min^−1^)	q_e_ (calc.) (mg g^−1^)	q_e_ (Exp.) (mg g^−1^)
	0.306	23.95	2.290	3.21
2nd-order-kinetic model	R^2^	K_2_ × 10^3^ (g mg^−1^ min^−1^)	q_e_ (calc.) (mg g^−1^)	q_e_ (Exp.) (mg g^−1^)
	0.999	102.10	3.81	3.21
Film-diffusion-kinetic model	R^2^	K_DF_		
	0.228	0.0019		

**Table 6:** Thermodynamic parameters for the adsorption of IV2R ions onto *P. capillacea* nanoparticles.

∆H^O^ (kJ mol^−1^)	∆S^O^ (J mol^−1^ K^−1^)	∆G^O^ (kJ mol^−1^)				
		25 °C	30 °C	40 °C	50 °C	60 °C
−27.82	0.117	−5.5586	−5.80675	−12.9078	−14.3678	−6.9785

**Table 7:** Comparison between sorption capacities of different pollutants onto different seaweed species.

Seaweed Species	Seaweed Family	pH	Sorption Capacity (mg g^−1^)	Refs.
*Ulva lactuca*	Green	5.5	29.069	[11]
*Caulerpa lentillifera*	Green	7	417	[93]
*Sargassum latifolium*	Brown	6&7	0.276	[94]
*Nizamuddinia zanardinii*	Brown	6.5	565.96	[95]
*Polysiphonia lanosa*	Red	2	45.8	[96]
*Palmaria palmate*	Red	2	33.8	[96]
*Gracilaria parvispora*	Red	8.0	77.18	[95]
*Pterocladia capillacea*	Red	6	49.50	[2]
*Pterocladia capillacea* nanoparticles	Red	2	100	current study

## Data Availability

The data that support the findings of this study are available from the authors upon reasonable request.
